# Dependency of solvation effects on metal identity in surface reactions

**DOI:** 10.1038/s42004-020-00428-4

**Published:** 2020-12-10

**Authors:** Mehdi Zare, Mohammad Saleheen, Subrata Kumar Kundu, Andreas Heyden

**Affiliations:** Department of Chemical Engineering, University of South Carolina, 301 Main Street, Columbia, South Carolina 29208 USA

**Keywords:** Heterogeneous catalysis, Biofuels, Computational chemistry

## Abstract

Solvent interactions with adsorbed moieties involved in surface reactions are often believed to be similar for different metal surfaces. However, solvents alter the electronic structures of surface atoms, which in turn affects their interaction with adsorbed moieties. To reveal the importance of metal identity on aqueous solvent effects in heterogeneous catalysis, we studied solvent effects on the activation free energies of the O–H and C–H bond cleavages of ethylene glycol over the (111) facet of six transition metals (Ni, Pd, Pt, Cu, Ag, Au) using an explicit solvation approach based on a hybrid quantum mechanical/molecular mechanical (QM/MM) description of the potential energy surface. A significant metal dependence on aqueous solvation effects was observed that suggests solvation effects must be studied in detail for every reaction system. The main reason for this dependence could be traced back to a different amount of charge-transfer between the adsorbed moieties and metals in the reactant and transition states for the different metal surfaces.

## Abstract (web summary)

Solvents play a central role in catalytic reactions, but predicting specific solvation effects in heterogeneous systems remains a challenge. Here, a hybrid quantum mechanical/molecular mechanical method is used to elucidate solvation effects on O–H and C–H bond cleavage in ethylene glycol over the (111) facet of six transition metals.

## Introduction

The widespread use of solvents in applications varying from pharmaceutical^1–6^ to electrochemistry^7–10^ and catalysis^11–16^ has given rise to an extensive range of studies aimed at understanding and predicting the role of solvents. The concept that a solvent can alter the performance of a catalyst, including its rate, selectivity, and stability, is well known; yet predicting a specific solvation effect remains a challenge^17–24^. While impressive progress has been made in understanding the effects of solvents in homogenous catalysis^25–29^, for heterogeneously catalyzed processes that benefit from easier separation of the catalyst relative to homogeneously catalyzed processes^30^, the role of solvents is hardly understood and only rarely studied. The inherent complexity of a reaction system containing both a complex heterogeneous catalyst and a condensed phase at a finite, often elevated, temperature has resulted in only few systematic experimental (in situ and in operando)^13,15,31,32^ and/or theoretical studies^11,12,33,34^ of solvation effects in heterogenous catalysis.

The role of solvents in catalytic transformations occurring at a solid-liquid interface is typically ascribed to: heightened importance of mass transfer effects, nature of solvent (polarity etc.)^35–37^, competitive adsorption between solvent molecules and adsorbed moieties^32,38,39^, direct participation of the solvent in the reaction coordinate^40^, and/or relative stabilization of reactant, transition and/or product state of elementary reactions^41–43^. These effects in turn can lead to a change in reaction mechanism, reaction kinetics, selectivity, and overall catalyst lifetime. In short, understanding and predicting solvent effects on surface reactions requires detailed investigations of the direct and indirect interactions between the solvent, catalyst, and reacting moieties on the surface under reaction conditions.

In this regard, theoretical calculations have the advantage of being able to systemically study the effect of an individual parameter on the effect of a solvent. Ab initio molecular dynamics (AIMD) simulations have been used^44–46^; however, due to the great computational cost associated with the quantum mechanical calculations and the large amount of phase space sampling necessary, AIMD simulations are currently limited to simulation systems of a few hundred atoms and a time scale of tens or a few hundred picoseconds^10,47,48^.

An alternative approach is to use implicit solvation models^49,50^. While they can compute free energies of reactions at solid-liquid interfaces rapidly, their reliability has often been questioned^14^ because of their inability to capture the anisotropic site-specific interactions between the solute and the solvent molecules. A compromise in efficiency and accuracy constitutes a combined quantum mechanical/molecular mechanical (QM/MM) approach^51–53^. In this class of simulations, the adsorbate and metal atoms involved in the reaction are considered as a QM sub-system described from first principles, while the bulk of the solvent and metal atoms distant to the active site are considered as an MM sub-system described using classical molecular mechanics force fields. We have previously developed such a hybrid QM/MM model, named *eSMS* (explicit solvation model for metal surfaces)^54^, which considers the long-range electrostatic interaction of the solvent molecules in the electronic structure calculation of the active site and applied it to the free energy calculation of the initial dehydrogenation and dehydroxylation of an adsorbed ethylene glycol (EG) moiety on Pt(111) in the presence of liquid water^55^.

To design a liquid-phase surface-catalyzed reaction system for enhanced activity and selectivity, the interrelation of energetic changes on variation of catalyst surface, nature of solvent, and reacting moiety must be disclosed. In this context, several studies have been dedicated to understanding how the nature of the solvent and reacting moiety direct catalysis^23,26,56–58^. Nonetheless, the role of the metal identity (catalyst) on the solvent effect has to our knowledge not been investigated yet. While it could be argued that solvation effects should be similar for the same bond cleavages or for the same adsorbates on different metal surfaces such as in recent studies by Greely et al.^59,60^, we hypothesize that the electronic structure modification of the metal surface and reacting moiety as a result of the nearby solvent is sufficiently significant that solvation effects can differ significantly for different metals. To confirm this hypothesis, we have investigated the aqueous-phase effects on the initial C–H and O–H bond cleavages of EG over the (111) facet of six transition metal surfaces (Ni, Pd, Pt, Cu, Ag, Au) using our explicit solvation method, *eSMS*. The choice of reaction systems is motivated by (i) EG being a commonly studied surrogate molecule of biomass-derived polyols, (ii) the selected transition metals are relatively stable and commonly used for aqueous-phase processing of biomass-derived oxygenates^61^, (iii) early dehydrogenation steps of EG over Pt and Ni/Pt catalysts have previously been found to control the overall reaction rate^62–64^, (iv) at least over Pt(111) in the vapor phase, initial C–H and O–H bond cleavage are competitive (although O–H bond cleavage is believed to be somewhat favored)^34,64^, and finally (v), explicit solvation approaches have recently been used to demonstrate that for bond cleavage reactions of alcohols over Pt(111), aqueous solvation effects are large and can currently not be described by implicit solvation models^55,65^.

## Results

**Fig. 1:**
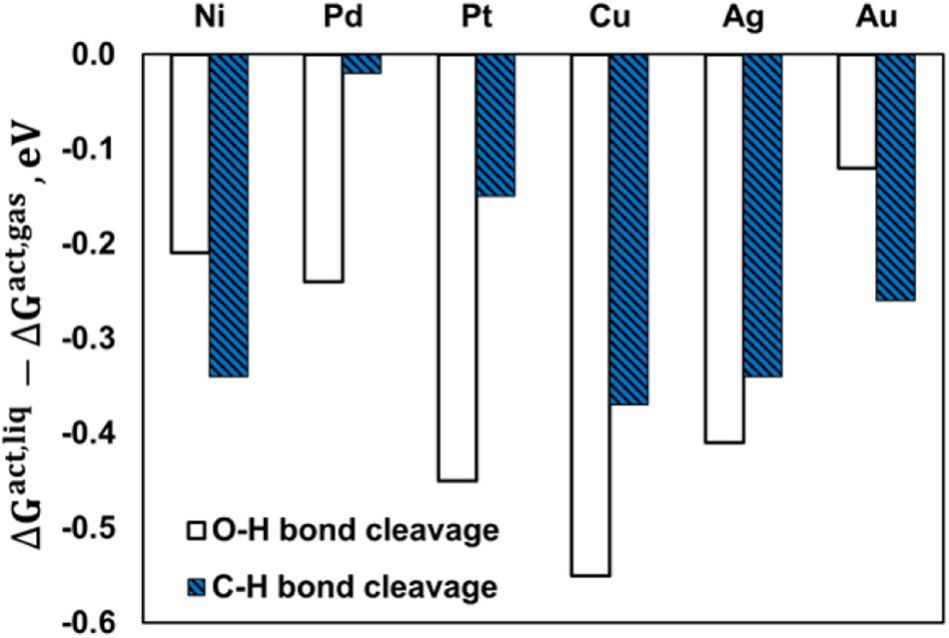
Aqueous-phase effects on the activation free energy barriers by eSMS. The investigated reactions include the O–H and C–H bond cleavages of ethylene glycol over the (111) surface facet of six transition metals at 423 K (all structures optimized in liquid water; see Table 1 for specific numbers).

**Fig. 2:**
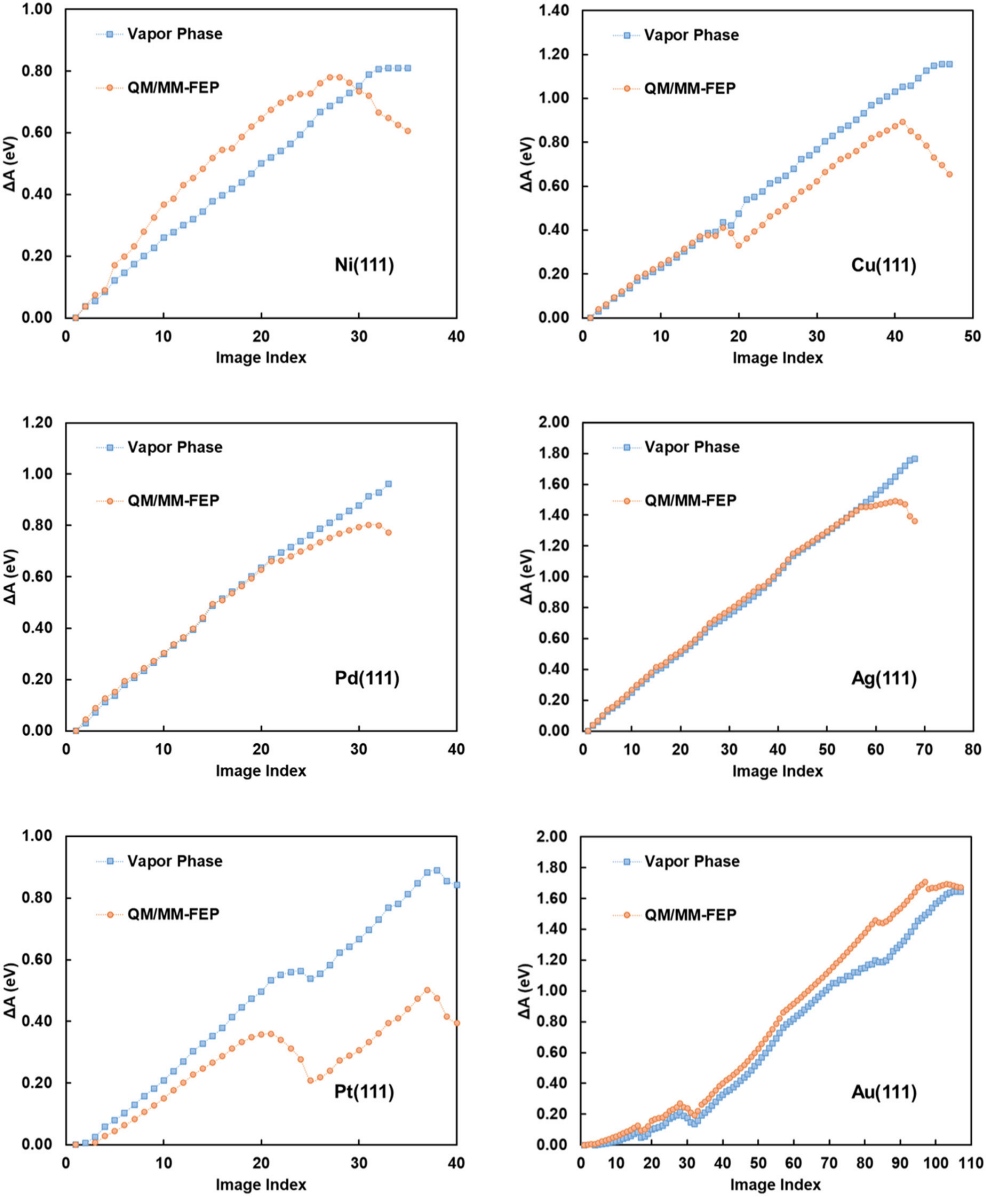
Free-energy profiles for O–H bond cleavage of ethylene glycol at 423 K. The profiles are for vapor and aqueous phase over the (111) surface facets of six transition metals without considering vibrational contributions to the partition functions. See Table 1 for corresponding data that include vibrational contributions. The number of intermediate states between the reactant and transition states for eSMS calculations is determined by our desire to have an energy difference between intermediate states smaller than twice the thermal energy (<2*k*_*B*_*T*). The aqueous-phase profiles portray the average of three or more independent eSMS calculations possessing 95% confidence intervals smaller than ±0.05 eV (see Table 1). The analogous plot for C–H bond cleavage reaction is provided in the Supplementary Fig. 1.

Figure 1 illustrates the aqueous-phase effects on the activation free energy barrier of the O–H (CH_2_OHCH_2_OH** + * ↔ CH_2_OCH_2_OH** + H*) and C–H bond cleavages (CH_2_OHCH_2_OH** + * ↔ CHOHCH_2_OH ** + H*) of EG at 423 K computed by *eSMS* (see Table 1 for specific numbers). In addition, a graphical representation of the free-energy (potential of mean force) profiles for the O–H bond cleavages is illustrated in Fig. 2 and for the C–H bond cleavages in Supplementary Fig. 1. We note that although some of the metals (such as Ni) might get partially oxidized in liquid water environments with low reduction potential, we chose to study the (111) facet of all metal surfaces for better comparison. Generally, both O–H and C–H bond cleavages are somewhat facilitated in the presence of water. For the O–H bond cleavage, Pt is the most active catalyst in both phases. However, for the C–H bond cleavage, Ni is the most active catalyst in the presence of liquid water because of strong aqueous-phase effect that is more than twice as large over Ni than Pt. Interestingly, in liquid water Cu is predicted to be as active for O–H bond cleavage (Δ*G*^act,liq^ = 0.50 eV) as Ni (Δ*G*^act,liq^ = 0.55 eV) and Pd (Δ*G*^act,liq^ = 0.58 eV). In short, evidenced by very different aqueous-phase effects on the activation free energy barriers across different metals, our hypothesis that the nature of metal plays a key role for solvation effects on surface reactions has been confirmed.

**Table 1:** Free energies of activation of O–H bond cleavage (CH_2_OHCH_2_OH** + * ↔ CH_2_OCH_2_OH** + H*) and C–H bond cleavage (CH_2_OHCH_2_OH** + * ↔ CHOHCH_2_OH ** + H*) of ethylene glycol in gas- and aqueous-phase environments over the (111) surface facet of six transition metals at 423 K.

Metal	Reaction Environment	O–H cleavage	C–H cleavage
Ni(111)	Vapor Phase	0.70	0.79
	VASPsol	0.67	0.73
	*iSMS*	0.62	0.75
	QM/MM-FEP	0.49 ± 0.06	0.45 ± 0.00
	QM/MM-MFEP-OPT	0.55 ± 0.06	0.43 ± 0.02
Pd(111)	Vapor Phase	0.83	0.76
	VASPsol	0.85	0.68
	*iSMS*	0.86	0.75
	QM/MM-FEP	0.59 ± 0.00	0.74 ± 0.02
	QM/MM-MFEP-OPT	0.58 ± 0.00	0.76 ± 0.02
Pt(111)	Vapor Phase	0.68	0.71
	VASPsol	0.82	0.64
	*iSMS*	0.62	0.62
	QM/MM-FEP	0.23 ± 0.01	0.56 ± 0.01
	QM/MM-MFEP-OPT	0.25 ± 0.01	0.55 ± 0.01
Cu(111)	Vapor Phase	1.08	1.43
	VASPsol	1.01	1.31
	*iSMS*	0.99	1.31
	QM/MM-FEP	0.52 ± 0.04	1.06 ± 0.00
	QM/MM-MFEP-OPT	0.50 ± 0.03	1.02 ± 0.00
Ag(111)	Vapor Phase	1.63	1.91
	VASPsol	1.65	1.81
	*iSMS*	1.57	1.88
	QM/MM-FEP	1.22 ± 0.04	1.57 ± 0.03
	QM/MM-MFEP-OPT	1.14 ± 0.04	1.56 ± 0.03
Au(111)	Vapor Phase	1.67	1.69
	VASPsol	1.75	1.43
	*iSMS*	1.64	1.52
	QM/MM-FEP	1.55 ± 0.00	1.43 ± 0.01
	QM/MM-MFEP-OPT	1.51 ± 0.01	1.41 ± 0.03

QM/MM-FEP indicates a free energy calculation in water between the critical points identified by gas-phase calculations (using the gas-phase vibrational partition function for the reactant and transition states). QM/MM-MFEP-OPT represents a free energy calculation in water for the different cleavages between the respective reactant and transition states that have been optimized in an aqueous-phase environment. Here, the vibrational partition functions are computed in the aqueous phase assuming the timescale for reorientation of the solvent molecules is much larger than the timescale for molecular vibrations. For comparison, implicit solvation calculations have also been performed using both nonperiodic (iSMS)^69^ and periodic (VASPsol)^70,71^ approaches. All numbers are in eV.

### Origin of dependency of solvent effect on metal identity

A heterogeneously catalyzed reaction occurring at a catalyst-solvent interface is at least a three-body problem involving solvent, catalyst, and reacting moiety. Thus, the most important factors contributing to solvation effects in such catalytic transformation arise from direct and indirect interactions of solvent, catalyst, and reacting moiety. To explain the origin for the variability of aqueous-phase effects on the free energy of activation (ΔΔ*G*^act^ = Δ*G*^act,liq^ − Δ*G*^act,gas^) of O–H and C–H bond cleavages of EG across six transition metals, we investigated some intuitive, physics-based descriptors.

One possible descriptor that could explain the role of the metal identity, which leads to different ΔΔ*G*^act^ values across different metals and bond cleavages, is the difference in charge-transfer effects (and thus solvent stabilization) in the reactant (RS) and transition states (TS) (see Supplementary Table 5). This charge transfer effect is attributed to the indirect influence of the water environment causing an electronic modification of the metal atoms and the effective charge distribution in the reacting moieties. We note that, using AIMD simulations, Siemer et al.^17^ have recently shown that water induced local charge transfer is a leading contribution for the observed solvation effect on O_2_ activation at Au/TiO_2_ interface sites. We choose two suitable descriptors for quantifying this charge-transfer effect. One is the cleaving-bond charge transfer (BC) defined as the change in sum of partial NPA^66^ charges on the cleaving bond going from RS to TS; for example in O–H bond cleavage: ${\rm{BC}} = \left| {Q^{\rm{O}} + Q^{\rm{H}}} \right|^{{\rm{RS}}} - \left| {Q^{\rm{O}} + Q^{\rm{H}}} \right|^{{\rm{TS}}}$. The idea behind this formula is that a change in charge from, e.g., −0.1 to +0.1, +0.1 to +0.1, or −0.1 to −0.1, should all result in no significant net stabilization since water stabilizes the RS and TS similarly. Another descriptor describing a charge-transfer effect is the molecular charge transfer (MC) defined here as the change in absolute sum of charges on the reacting moiety going from RS to TS, i.e., this descriptor describes the change in charge transfer from the metal surface to the reacting moiety when going from the RS to the TS.

Table 2 lists BC and MC for the O–H and C–H bond cleavages over the (111) facet of six transition metal surfaces (see Supplementary Table 5 for partial charges). According to our definition of BC and MC, smaller BC values (more negative) or larger MC values (more positive) correspond to an increased charge-transfer effect when going from reactant to transition state. For instance, in the C–H bond cleavage, the charge-transfer effect over Cu(111) (BC = −0.46 e, MC = 0.53 e) is higher than over Pt(111) (BC = −0.03 e, MC = 0.08 e). As shown in Table 2, ΔΔ*G*^act^ is directly related to the charge-transfer effect descriptors in the C–H bond cleavage; that is, solvent effects increase (more negative ΔΔ*G*^act^) with increasing charge-transfer effect (more negative BC or more positive MC). In contrast, for O–H bond cleavage, there is no linear relationship between ΔΔ*G*^act^ and the charge-transfer effect. Nevertheless, after investigating other solvation effect descriptors, we will show that the charge-transfer effect is likely also here a key descriptor for the solvent effect across metal surfaces.

**Table 2:** Average aqueous-phase effects on the activation free energy (ΔΔ*G*^act^ = Δ*G*^act,liq^ − Δ*G*^act,gas^) as well as three solvation effect descriptors for the O–H and C–H bond cleavages of ethylene glycol over the (111) surface facet of six transition metals at 423 K (see Table 1 for 95% confidence intervals).

Cleavage	Surface	ΔΔ*G*^act^, eV	Δ*G*^act,gas^, eV	H-bond	MC, e	BC, e
O–H	Ni	−0.21	0.70	0.99	0.64	−0.61
	Pd	−0.24	0.76	0.34	0.13	−0.36
	Pt	−0.45	0.68	0.54	−0.10	−0.24
	Cu	−0.55	1.08	0.77	0.69	−0.74
	Ag	−0.41	1.63	0.92	0.78	−0.77
	Au	−0.12	1.67	0.95	0.62	−0.63
C–H	Ni	−0.34	0.79	0.26	0.41	−0.36
	Pd	−0.02	0.83	−0.26	−0.03	0.06
	Pt	−0.15	0.71	0.12	0.08	0.03
	Cu	−0.37	1.43	0.21	0.53	−0.46
	Ag	−0.34	1.91	−0.27	0.28	−0.32
	Au	−0.26	1.69	−0.38	0.06	−0.12

Δ*G*^act,gas^ is the free energy of activation in vapor phase, H-bond denotes the change in mean of total hydrogen bonding (acceptor + donor) going from RS to TS, MC (molecular charge transfer) represents the change in the absolute sum of partial charges on the reacting moiety going from RS to TS, and finally BC (cleaving-bond charge transfer) is the change in sum of partial charges on the cleaving bond atoms (see Supplementary Table 5 for partial charges) going from RS to TS.

Another commonly used criterion for describing adsorbate-solvent interaction in an aqueous phase is hydrogen bonding^23,41^. Two main classes of hydrogen bonding definitions commonly used in the literature are based on an energy criterion and a geometric definition. Herein, we employed a geometric definition in which a hydrogen bond exists if the distance between the donor oxygen (O_d_) and the acceptor oxygen (O_a_), *R*_OO_, is less than 3.2 Å and the angle ∠HO_d_O_a_ is smaller than 20°^67^ (see Supplementary Fig. 2). We note that EG with OH functional groups can be either a donor or acceptor of hydrogen bonding (Supplementary Table 4). Hence, the change in mean of total hydrogen bonding (acceptor + donor) going from RS to TS was chosen as a descriptor, named in the following H-bond, and is included in Table 2. Next, the gas-phase free energy of activation (Δ*G*^act,gas^), a rough measure of change in surface-adsorbate interaction going from reactant to transition state was selected as a descriptor for describing the variability in solvation free energy effects across metal surfaces. Given the absence of a linear ΔΔ*G*^act^ dependence on H-bond or Δ*G*^act,gas^, we also studied all pairwise combinations of descriptors. Finally, we note here that we also analyzed the water orientation (H-up, H-down, parallel – see Supplementary Fig. 3) within the first water layer of the different surfaces in the reactant state (see height distribution function of water O in Supplementary Fig. 4); however, we did not observe any significant variation in water orientations across metals, explaining why we did not further study water orientation as a descriptor (Supplementary Table 10). Also, we attempted to use the standard electrode potential of the metal elements as descriptor but again no meaningful correlation could be obtained such that it is not further discussed.

Next, we first examined the pairwise correlations between the descriptors using the Pearson correlation coefficient (PCC)^68^. The results (see Supplementary Table 6) indicate that BC and MC are totally correlated (PCC ~ −1.0), which is expected given that they both describe charge transfer. In addition, H-bond is more correlated with MC and BC in the O–H bond cleavage than in the C–H bond cleavage. We attribute this to the fact that hydrogen bonding is obtained from the interaction of water molecules with the OH functional groups of EG, and the O–H bond cleavage reaction causes a significant change in the charges on one of the two O–H functional groups in EG in the TS. Finally, Δ*G*^act,gas^ is hardly correlated with BC, MC or H-bond in both cleavages.

Second, using a simple linear model (Eq. 1), we investigated the relation between the descriptors and the aqueous-phase effects on the free energies of activation (ΔΔ*G*^act^). We emphasize that we do not intend to quantitatively predict ΔΔ*G*^act^ using a set of descriptors. Instead, we employ models based on physics-based descriptors to explain what physical phenomena could explain the changes in solvation effects on the kinetics of the studied surface-catalyzed reactions.

1 $$(\Delta \Delta G^{{\rm{act}}} - \overline {\Delta \Delta G^{{\rm{act}}}} )_{{\rm{model}}} = \alpha _1(f_1 - \overline {f_1} ) + \alpha _2(f_2 - \overline {f_2} )$$

In eq. 1, bar signs show the mean of the corresponding variable, *α*_1_ and *α*_2_ are model parameters, and *f*_1_ and *f*_2_ represent descriptors (Table 2). The best-fitting parameters and mean absolute error (MAE) of the linear model for different combinations of descriptors are listed in Supplementary Table 7. Interestingly, the linear model can estimate ΔΔ*G*^act^ very well for the C–H bond cleavage but not for the O–H bond cleavage. As expected, the dominant factor in C–H bond cleavage is the charge-transfer effect; that is, $\alpha _2 \gg \alpha _1$ when one of charge-transfer effect descriptors (BC or MC) used as *f*_2_ (see Supplementary Table 7). Finally, a quadratic model with two descriptors was employed to explain ΔΔ*G*^*act*^ of the O–H bond cleavage.

2 $$(\Delta \Delta G^{{\rm{act}}} - \overline {\Delta \Delta G^{{\rm{act}}}} )_{{\rm{model}}} =\,	 \alpha _1(f_1 - \overline {f_1} ) + \alpha _2(f_2 - \overline {f_2} ) \\ 	+ \alpha _3(f_1 - \overline {f_1} )^2 + \alpha _4(f_2 - \overline {f_2} )^2$$

The results of this model for different combinations of descriptors are included in Supplementary Table 8.

**Fig. 3:**
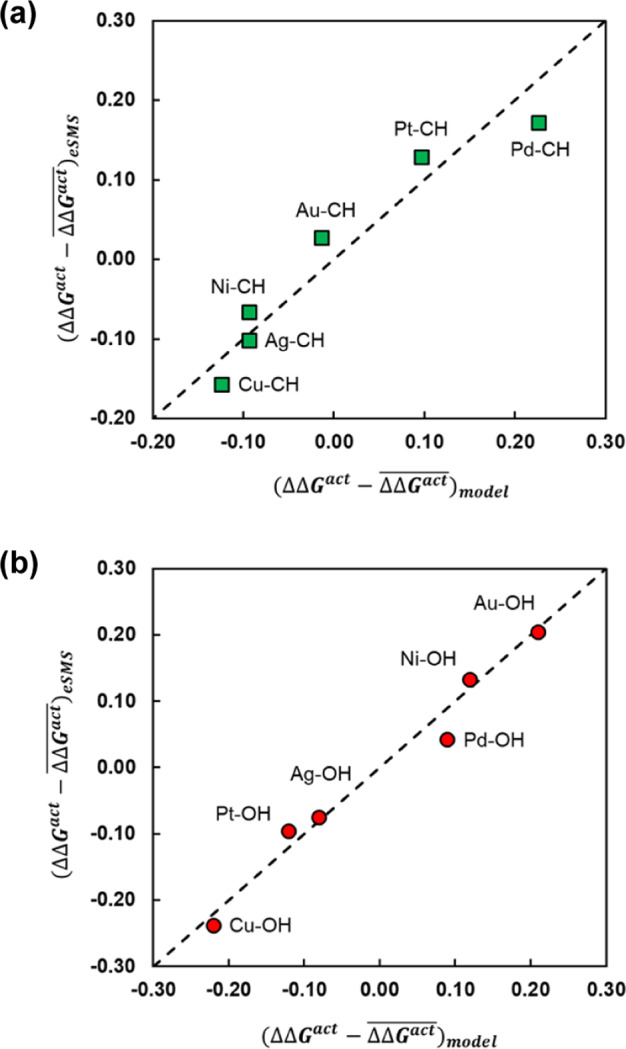
Parity plot of the aqueous-phase effect on the activation free energy. The plot compares the aqueous-phase effect on activation free energy (ΔΔ*G*^act^ = Δ*G*^act,liq^ − Δ*G*^act,gas^) computed by eSMS (QM/MM-FEP in Table 1) against the model-predicted aqueous-phase effect. The predicted values are calculated using the gas-phase free energy of activation (Δ*G*^act,gas^) and cleaving-bond charge transfer (BC) as two descriptors in (**a**) a linear model (eq. 1) for C–H bond cleavage and (**b**) a quadratic model (eq. 2) for O–H bond cleavage of ethylene glycol over the (111) surface facets of six transition metals at 423 K. The average aqueous-phase effect, $\overline {\Delta \Delta G^{{\rm{act}}}}$, computed by eSMS, is −0.33 eV for O–H bond cleavage and −0.25 eV for C–H bond cleavage. The mean of absolute errors (MAE) between eSMS and model-predicted values is 0.03 eV in the linear model (**a**) and 0.02 eV in the quadratic model (**b**). The corresponding model parameters are listed in Supplementary Tables 7 and 8.

Overall, when comparing the model parameters of the linear and quadratic fits, we conclude that the aqueous-phase effect on the free energy of activation of O–H and C–H bond cleavages of EG over the investigated metal surfaces originates primarily from some form of a charge-transfer effect (BC or MC) (more charge transfer in the TS relative to the RS leads to more stabilization) and partly on another descriptor that could be related to H-bond and Δ*G*^*act,gas*^ (larger barriers are more stabilized). Figure 3 presents the computed aqueous-phase effect by *eSMS* versus the estimated one from a linear fit for C–H bond cleavage and a quadratic fit for O–H bond cleavage, in which BC and Δ*G*^act,gas^ were used as descriptors.

### Comparison to implicit solvation models

Finally, the implicit solvation models (iSMS^69^ and VASPsol^70,71^) failed to capture the full solvent stabilization during the O–H bond cleavage of EG (see Table 1). For the C–H bond cleavage, implicit and explicit solvation models anticipate comparable aqueous-phase effects for the metals that display a smaller charge transfer effect (Pd, Pt, Au). Supplementary Table 9 illustrates that this observation holds even when considering typical uncertainties in cavity radius of ±10% for transition metal elements. In short, the reliability of implicit solvation calculations for heterogeneous (metal) catalysis applications is currently limited (unknown) due to the very limited availability of experimental data that can be used in the parameterization of the implicit solvation models. This appears to be currently an advantage for explicit solvation models that rely “only” on a meaningful potential energy description.

## Discussion

In summary, we hypothesized that since solvents can modify the electronic structure of a metal surface, thereby affecting the stability of reacting moieties, the metal identity plays a significant role for solvation effects on elementary reactions. We investigated aqueous-phase effects on the free energy of activation of the initial O–H and C–H bond cleavages of ethylene glycol over the (111) surface of six transition metals, including Ni, Pd, Pt, Cu, Ag, and Au, to disclose the role of metal identity on solvation effects. To compute the free energy of activation in the presence of water, we utilized our explicit solvation approach, named *eSMS*, which is based on a hybrid quantum mechanical/molecular mechanical (QM/MM) description of the potential energy surface. Our hypothesis was confirmed by finding significantly different aqueous-phase effects on the activation barrier (ΔΔ*G*^act^ = Δ*G*^act,liq^ − Δ*G*^*act,gas*^) for the same bond cleavage across different metals, suggesting that solvation effects have to be studied in detail for any specific reaction system. Subsequently, by introducing three intuitive, physics-based descriptors, the charge-transfer effect, hydrogen bonding, and the gas-phase activation free energy barrier, and studying correlations between the aqueous-phase effects and these descriptors, we can conclude that the aqueous-phase effects originate primarily from various charge-transfer effects across the different metals, and to a lesser extent to another descriptor that could be related to a different amount of hydrogen bonding and the gas-phase activation barriers across the different metals. This observation agrees with previous AIMD simulations of oxygen activation over Au catalysts^17^. Finally, implicit solvation models are currently not able to capture these charge-transfer effects that can lead to changes in preference of initial O–H versus C–H bond cleavage in alcohols over various transition metal surfaces.

## Methods

### Planewave DFT calculations

Vapor-phase DFT calculations were carried out using periodic boundary conditions as implemented in the Vienna Ab Initio Simulation Package (VASP 5.4.4)^72,73^. A frozen-core, all-electron projector augmented-wave (PAW)^74^ method was utilized to avoid the singularities of Kohn–Sham wavefunctions at the nuclear positions. The number of valence electrons considered for Ni, Pd, Pt, Cu, Ag, Au, C, O, and H are 10, 10, 10, 11, 11, 11, 4, 6, and 1, respectively. The unknown part of interaction energy between individual electrons, i.e., the exchange-correlation functional, was expressed using the Perdew–Burke–Ernzerhof (PBE)^75,76^ functional within the semi-local generalized gradient approximation^77^. Brillouin zone integrations have been performed with a 4 × 4 × 1 Monkhorst–Pack^78^ *k*-point grid and electronic wavefunctions at each *k*-point were expanded using a discrete plane-wave basis set with kinetic energies limited to 400 eV. A first order smearing method (Methfessel–Paxton)^79^ with 0.10 eV smearing width was employed, allowing to accurately calculate the entropic contributions due to the smearing. Dipole and quadrupole corrections (along the surface normal) to the total energy have been calculated using a modified version of the Makov–Payne^80^ method, and Harris corrections, based on the non-self-consistent Harris–Foulkes^81,82^ functional, have been applied to the stress-tensor and forces. A 4 × 4-unit cell with four layers of metal atoms (bottom two layers fixed in their bulk positions) has been employed. By introducing a 15 Å vacuum on top of the surface, the interaction between the periodic images along the surface normal has been curtailed. A self-consistent field (SCF) convergence criterion for the electronic degrees of freedom of the valence electrons was set to 1.0 × 10^−7^ eV. Transition state structures for the elementary processes were located using a combination of climbing-image nudged elastic band^83,84^ and dimer^85,86^ methods. Finally, the minima and the first order saddle points were validated by computing the Hessian matrix and vibrational spectra. We note that spin-polarized calculations have been carried out for the Ni surface.

### Non-periodic cluster calculations

Cluster model DFT calculations in vacuum have been carried out using the TURBOMOLE 7.2 program package^87–89^. To model the cluster surfaces, two layers of metal atoms with a hexagonal shaped geometry (51 atoms) were chosen. The convergence of the total QM/MM energy with respect to the lateral size and depth of the cluster geometry can be found elsewhere^54^. An improved version of the default TURBOMOLE basis sets (def-bases) with split valence and polarization functions (def2-SVP)^90,91^ were employed to represent the adsorbate atoms and the metal atoms of Ni and Cu. Furthermore, Ag, Pd, Pt, and Au atoms were represented using scalar relativistic effective core potentials (ECPs) in conjunction with split valence basis sets augmented by polarization functions^91,92^. Electron exchange and correlation effects were accounted for by employing the PBE functional^75,76^. To speed up the calculation as recommended by TURBOMOLE, the RI-J approximation with auxiliary basis sets was used to approximate the coulomb integrals^93,94^. An SCF convergence criterion of 1.0 × 10^−7^ Hartree was established and a Gauss-Chebyshev type spherical grid, m4, was employed to perform the numerical integrations^88^.

### Molecular dynamics (MD) simulations

MD simulations were carried out using the DL_POLY 4.03 molecular simulation program package^95^. The initial 4 × 4 unit cell for each metal surface was augmented laterally to a 16 × 20 surface with further vacuum added in the Z-direction resulting in a simulation box comprising of 1280 metal atoms. The simulation box size for each metal is reported in Supplementary Table 1. The simulation box height was selected based on the work from Behler et al.^67^ finding that simulations of metal-water interfaces should contain a water layer of ~40 Å height. The experimental saturated liquid water density of ~0.9 g/cm^3^ at 423 K was achieved by packing the simulation box of Ni, Pd, Pt, Cu, Ag, and Au with 2258, 2758, 2800, 2398, 3045, and 2985 water molecules, respectively. All metal and adsorbate atoms were kept fixed while the geometry of water molecules was constricted to that of TIP3P^96^ geometry with the RATTLE algorithm^97^. To solve the Newton’s equations of motion, a velocity version of the SHAKE algorithm^98^, in conjunction with the velocity Verlet (VV) integrator^99^ were used. The TIP3P model was employed for the force field parameters of liquid water while the van der Waals parameters for adsorbate atoms were obtained from the OPLS force field^100,101^. In addition to the OPLS parameters, the Lennard-Jones parameters from the Combined B3LYP/6-31_G*/AMBER Potential^102^ were used for the hydrogen atoms of the adsorbed moieties. Lennard-Jones parameters for hydrogen atoms are important in QM/MM optimizations that permit hydrogen atoms to approach water molecules and leave the protective environment of a neighboring carbon or oxygen atom. The Lennard-Jones metal potential^103^ was employed to describe the metal-water interaction. The LJ cross-term of intermolecular parameters were calculated by Lorentz-Berthelot mixing rules through equations $\sigma _{ij} = \frac{{\sigma _i + \sigma _j}}{2}$ and $\varepsilon _{ij} = \sqrt {\varepsilon _i\varepsilon _j}$. All Lennard-Jones parameters are included in Supplementary Table 2. The charges for the QM atoms were estimated using the natural population analysis (NPA)^66^. To describe the interaction of the TIP3P water point charges with the quantum chemically described cluster model, we employed the periodic electrostatic embedded cluster method (PEECM)^104^ as implemented in TURBOMOLE. Simulations were carried out in a canonical ensemble (NVT) with Nosé-Hoover thermostat^105,106^. A 1 ps relaxation time constant for temperature fluctuations was used to maintain the average system temperature. Electrostatic interactions were accounted for by using the Smoothed Particle Mesh Ewald (SPME) method^107^ with automatic parameter optimization for default SPME precision and a 12 Å cutoff radius was adopted for the van der Waals interactions and the transition between short and long-range electrostatic interactions. Unless specified otherwise, for each free energy perturbation step, all systems were equilibrated for 250 ps and sampled for 1000 ps (1ns) using a 1 fs timestep to obtain 1000 MM conformations (1 ps apart). Thus, MD simulations for over 2.9 ms were performed for this study. To optimize structures in an aqueous reaction environment, we utilized the fixed-size ensemble approximation with 5000 MM conformations (250 ps equilibration and 5 ns sampling) recorded every 1 ps.

### QM/MM energy calculation

The QM/MM minimum free energy path (QM/MM-MFEP)^52,53^ method for optimizing the intrinsic reaction coordinate on a potential of mean force (PMF) description of the reaction system has been implemented in our program packages. A full description of this methodology, *eSMS* (Explicit Solvation for Metal Surfaces) can be found elsewhere^54^. Briefly, the total energy function formulation of our *eSMS* method is given by

3 $$\begin{array}{ccccc}\\ E_T\left( {\underline r _{{\rm{QM}}},\underline r _{{\rm{MM}}}} \right) = E_{{\rm{QM}}}^{{\rm{Surface}}}\left( {\underline r _{{\rm{QM}}}} \right) - E_{{\rm{QM}}}^{{\rm{cluster}}}\left( {\underline r _{{\rm{QM}}}} \right) + \left\langle \Psi \right|H_{{\rm{eff}}}\left( {\underline r _{{\rm{QM}}},\underline r _{{\rm{MM}}}^{{\rm{MeanField}}\left( {100} \right)}} \right)\left| \Psi \right\rangle \\ \\  + \left[ {\frac{1}{{100}}\mathop {\sum}\limits_{j = 1}^{100} {\left[ {E_{j,{\rm{MM}} + {\rm{QM}}/{\rm{MM}}}^{{\rm{elec}} + {\rm{vdW}}}\left( {\underline r _{{\rm{QM}}}\underline r _{MM}^{MeanField\left( {100} \right)}} \right)} \right]} } \right]_{Q_i = 0,i \in QM} \\ \\ - E_{j,{\rm{MM}} + {\rm{QM}}/{\rm{MM}}}^{{\rm{elec}} + {\rm{vdW}}}\left( {\underline r _{{\rm{QM}}}\underline r _{{\rm{MM}}}^{{\rm{MeanField}}\left( {100} \right)}} \right) +\, E_{j,{\rm{MM}} + {\rm{QM}}/{\rm{MM}}}^{{\rm{elec}} + {\rm{vdW}}}\left( {\underline r _{{\rm{QM}}},\underline r _{{\rm{MM}}}} \right)\\ \end{array}$$

where the first term is evaluated for a periodic slab using the VASP program package (*planewave DFT calculation*), the second term is a QM cluster calculation in vacuum computed with the TURBOMOLE program package (*non-periodic cluster calculations*), and the third term is a QM cluster calculation in a periodic mean field of MM water molecules computed using the periodic electrostatic embedded cluster method (PEECM) in TURBOMOLE under the fixed-charge approximation (fixed-charge approximation has been validated for our *eSMS* approach^54^). We note that the number 100 in the equation indicates that 100 MM conformations, selected equally spaced from equilibrated 1 ns molecular dynamic (MD) simulations (10 ps apart), were used to represent the mean field of the MM water molecules. Finally, the last three terms account for the classical (MM level of theory) electrostatic and van-der-Waals interaction energy of the total system without overcounting the electrostatic interaction of the MM (water) molecules with the QM cluster subsystem. We note that all solvent (water) molecules are described in this study at the MM level of theory.

Free energy calculations require energy evaluation from uncorrelated measurements of the system and ideally the energy estimator should also be capable of minimizing the statistical bias and variance of the free energy differences of the physical system being studied. Exponential averaging (EXP), also known as the Zwanzig relationship^108^ has long been applied to study a variety of problems such as amino acid recognition^109^, RAS-RAF binding affinity^110^, and octanol/water partition coefficients^111^, etc. However, the EXP has been shown to represent poor efficiency and phase space overlap^112,113^, and is also largely dependent on the distribution of the QM/MM energy^114^. Here, we employed the Bennett Acceptance Ratio (BAR)^115^ as the free energy estimator which uses both the forward and reverse distributions simultaneously in a more efficient way than simply averaging the forward and reverse exponential estimators. BAR has been demonstrated to benefit from a lower bias and variance of the free energy estimates in practical atomistic simulations when compared to EXP and thermodynamic integration (TI)^112,116^. Finally, the whole free energy estimation procedure has been repeated three times independently to establish 95% confidence intervals for evaluating the free energy of activation, assuming a normal distribution^117^. More independent simulations were carried out only if these three experiments were not resulting in 95% confidence interval smaller than 0.05 eV. All uncertainties reported in this study are 95% confidence intervals.

### Average rotational correlation time

Adequately sampling of the potential energy surface for all relevant configurations of the system is of great importance in any QM/MM approach for computing the liquid-phase effect on the free energy of elementary processes^114^. Owing to a lack of consensus on how much sampling of the configurational space is sufficient for a solvated adsorbed carbohydrate species on a metal surface for an error smaller than 0.05 eV, we computed the average rotational correlation time for water molecules in close proximity (up to 5 Å) to adsorbed ethylene glycol in the reactant and transition states over the (111) facet of six transition metals and reported them in Supplementary Table 3. The longest average correlation time is computed to be ~200 ps. Hence, we decided to sample for 1000 ps to make sure that relevant configurations of the systems are sampled adequately. As discussed above, for free-energy calculations, the procedure was repeated at least three times with independent MD trajectories to establish the confidence interval of the computation of the free energy of activation in aqueous phase.

### Non-periodic implicit solvation calculations

The implicit solvation model for solid surfaces (iSMS)^69^ was utilized to compute the activation free energies in an aqueous reaction environment as

4 $$\Delta G_{{\rm{solvent}}}^\ddagger = \Delta G_{{\rm{Gas}}}^\ddagger + \left[ {G_{{\rm{solvent}}}^{{\rm{TS}}} - G_{{\rm{solvent}}}^{{\rm{IS}}}} \right]$$

where $\Delta G_{{\rm{Gas}}}^\ddagger$ is the respective activation free energy under gas phase conditions, and $G_{{\rm{solvent}}}^{IS}$ and $G_{{\rm{solvent}}}^{{\rm{TS}}}$ represent the solvation free energies of initial and transition states, respectively, computed as

5 $$G_{{\rm{surface}} + {\rm{intermediate}}}^{{\rm{liquid}}} = G_{{\rm{surface}} + {\rm{intermediate}}}^{{\rm{vacuum}}} + \left( {G_{{\rm{cluster}} + {\rm{intermediate}}}^{{\rm{liquid}}} - E_{{\rm{cluster}} + {\rm{intermediate}}}^{{\rm{vacuum}}}} \right)$$

where $G_{{\rm{surface}} + {\rm{intermediate}}}^{{\rm{vacuum}}}$ is the free energy of an intermediate (e.g., IS or TS) in the absence of a solvent, computed here within the harmonic approximation using plane-wave DFT calculations for periodic slab models, $G_{{\rm{cluster}} + {\rm{intermediate}}}^{{\rm{liquid}}}$ is the free energy of the metal cluster and surface intermediate in liquid without vibrational contributions (that are already considered in the first term), and $E_{{\rm{cluster}} + {\rm{intermediate}}}^{{\rm{vacuum}}}$ is the DFT energy of the same cluster in the absence of the solvent. To compute the $G_{{\rm{cluster}} + {\rm{intermediate}}}^{{\rm{liquid}}}$ term, COSMO-RS^118,119^ (conductor-like screening model for real solvents) calculations are performed using the COSMOtherm program with latest FINE parameterization^120^. The COSMOtherm program for solvent thermodynamic properties requires COSMO calculations to be performed at the BP-TZVPD level of theory. Given the uncertainty in cavity radius for transition metal elements, calculations are performed with default and 10% increased and decreased cavity radius for the transition metal elements (see Supplementary Table 9).

### Periodic Implicit Solvation Calculations

In addition to implicit solvation calculations performed with *iSMS* method, implicit solvation calculations were also performed with VASPsol^70,71^ using a relative permittivity of water of 44.07 at 423 K^121^. For VASPsol, we used the default values for the parameter *n*_*c*_ that defines the value at which the dielectric cavity forms and for the width of the diffuse cavity, *σ*, and for effective surface tension parameter, *τ*, describing the cavitation, dispersion, and repulsive interaction between the solute and the solvent that are not captured by the electrostatic terms^71^. While the parameters are likely most accurate only for simulations at 298 K and not at 423 K, they are the optimized parameters of the solvent model that cannot easily be obtained at other temperatures. Due to the absence of adequate experimental solvation data at 423 K, we decided that the default parameters are likely most meaningful, i.e., the relative permittivity is the only temperature dependent solvent parameter in our VASPsol model. All other computational details for periodic implicit solvation calculations were kept the same as in our periodic vapor-phase calculations.

## Supplementary information

Supplementary Information

Description of Additional Supplementary Files

Supplementary Data 1

## Data Availability

The authors declare that the data supporting the findings of this study are available within the paper and its supplementary information files. We note that the optimized atomic coordinates of reactant (RS) and transition states (TS) clusters in gas and liquid phase are provided in Supplementary Data 1 file.
