# Supplementary material for: Dependency of solvation effects on metal identity in surface reactions
Source: Commun Chem. 2020 Dec 10;3:187. doi: 10.1038/s42004-020-00428-4 (PMC9814277; doi:10.1038/s42004-020-00428-4)
Supplement: Supplementary file 1 — Supplementary Information [file 42004_2020_428_MOESM1_ESM.pdf]

Supplementary Information for the Paper:

Dependency of solvation effects on metal identity in surface reactions

Mehdi Zare, Mohammad Saleheen, Subrata Kumar Kundu, and Andreas Heyden*

Department of Chemical Engineering, University of South Carolina, 301 Main Street, Columbia, South Carolina 29208, USA

*Corresponding author: email: heyden@cec.sc.edu

Table of Contents

Tables 2

Figures 7

Tables

Supplementary Table 1. Simulation box size used for (111) facet of each metal surface.

Metal	Simulation box size, Å^3
Ni	39.75×43.03×49.01
Pd	44.53×48.20×49.01
Pt	44.98×48.69×49.01
Cu	41.04×44.43×49.01
Ag	46.85×50.71×49.01
Au	47.00×50.88×49.01

Supplementary Table 2. Lennard-Jones parameters of all elements used in this study. The parameters are based on 12-6 Lennard-Jones potential, $V_{lj} = 4\varepsilon\left[\left(\frac{\sigma}{r}\right)^{12} - \left(\frac{\sigma}{r}\right)^{6}\right]$. Ow and Hw represent oxygen and hydrogen of TIP3P water model, respectively.

Element	ε (kJ/mol)	σ (Å)
Ni	23.640	2.274
Pd	25.732	2.511
Pt	32.635	2.535
Cu	19.748	2.331
Ag	19.079	2.633
Au	22.133	2.629
C	0.495	3.911
O	0.710	3.071
H	0.126	1.978
Ow	0.636	3.151
Hw	0.192	0.400

Supplementary Table 3. Average rotational correlation time (in picosecond) of liquid water molecules residing in a 5 Å radius of an adsorbed ethylene glycol ($C_2H_5OH^*$) species on a (111) facet of six transition metal surfaces studied in the present work. The predicted correlation functions have been fitted with three exponential functions to acquire average rotational correlation times.

	**O-H cleavage**		**C-H cleavage**
**Surface**	RS	TS	TS
Ni	218	147	79
Pd	227	101	223
Pt	183	46	53
Cu	115	67	21
Ag	82	111	196
Au	73	43	32

Supplementary Table 4. Number of hydrogen bonds formed from the interactions of the TIP3P water model and the reacting moiety in reactant state (RS) and transition state (TS) of O-H and C-H bond cleavage of ethylene glycol over a (111) facet of six transition metal surfaces at 423 K. Acceptor indicates that OH functional groups of ethylene glycol accepts a hydrogen bond donated by the oxygen of water while Donor indicates vice versa. All numbers are computed based on three independent simulations and hence possess 95% confidence intervals (assuming a normal distribution).

	**O-H cleavage**				**C-H cleavage**			
	RS		TS		RS		TS	
**Surface**	Acceptor	Donor	Acceptor	Donor	Acceptor	Donor	Acceptor	Donor
Ni	1.50±0.08	0.34±0.06	2.66±0.13	0.17±0.03	1.50±0.08	0.34±0.06	1.96±0.06	0.14±0.04
Pd	1.23±0.08	0.63±0.10	2.14±0.10	0.05±0.04	1.23±0.08	0.63±0.10	1.37±0.05	0.22±0.03
Pt	1.17±0.10	0.11±0.02	1.82±0.04	0.00±0.00	1.17±0.10	0.11±0.02	1.23±0.10	0.17±0.02
Cu	1.47±0.08	0.35±0.03	2.47±0.12	0.12±0.05	1.47±0.08	0.35±0.03	1.79±0.12	0.24±0.03
Ag	1.51±0.11	0.40±0.05	2.81±0.10	0.01±0.00	1.51±0.11	0.40±0.05	1.38±0.06	0.26±0.02
Au	1.58±0.10	0.05±0.01	2.53±0.08	0.04±0.02	1.58±0.10	0.05±0.01	1.09±0.08	0.15±0.03

Supplementary Table 5. Partial charges (in atomic units), computed based on the NPA[1] charge model, on the reacting moiety (ethylene glycol) in the reactant (RS) and transition states (TS) for O-H and C-H bond cleavages over the (111) facet of six transition metal surfaces at 423 K. Bold numbers indicate partial charges on the cleaving bond atoms (O_1 and H_5 in O-H bond cleavage, and C_1 and H_4 in C-H bond cleavage).

Cleavage	Surface	State	C_1	C_2	O_1	O_2	H_1	H_2	H_3	H_4	H_5	H_6
**O-H**	Ni	RS	-0.11	-0.10	**-0.75**	-0.79	0.21	0.21	0.21	0.15	**0.45**	0.48
		TS	-0.11	-0.09	**-0.80**	-0.78	0.21	0.22	0.21	0.10	**-0.12**	0.47
	Pd	RS	-0.08	-0.10	**-0.75**	-0.72	0.21	0.20	0.22	0.17	**0.50**	0.47
		TS	-0.06	-0.11	**-0.71**	-0.71	0.23	0.18	0.22	0.15	**0.10**	0.47
	Pt	RS	-0.11	-0.10	**-0.75**	-0.71	0.22	0.23	0.22	0.19	**0.49**	0.51
		TS	-0.12	-0.06	**-0.68**	-0.69	0.24	0.14	0.23	0.20	**0.17**	0.48
	Cu	RS	-0.10	-0.10	**-0.75**	-0.79	0.21	0.21	0.20	0.17	**0.47**	0.48
		TS	-0.10	-0.10	**-0.86**	-0.78	0.19	0.20	0.21	0.21	**-0.16**	0.48
	Ag	RS	-0.10	-0.09	**-0.75**	-0.77	0.20	0.21	0.20	0.18	**0.48**	0.48
		TS	-0.11	-0.09	**-0.78**	-0.79	0.20	0.20	0.19	0.14	**-0.27**	0.47
	Au	RS	-0.13	-0.12	**-0.71**	-0.70	0.19	0.18	0.18	0.17	**0.48**	0.47
		TS	-0.13	-0.12	**-0.78**	-0.69	0.20	0.18	0.20	0.12	**-0.08**	0.48
**C-H**	Ni	RS	**-0.11**	-0.10	-0.75	-0.79	0.21	0.21	0.21	**0.15**	0.45	0.48
		TS	**-0.23**	-0.12	-0.73	-0.77	0.22	0.20	0.22	**-0.17**	0.45	0.48
	Pd	RS	**-0.08**	-0.10	-0.75	-0.72	0.22	0.20	0.22	**0.17**	0.50	0.47
		TS	**-0.06**	-0.12	-0.75	-0.68	0.25	0.21	0.23	**0.03**	0.50	0.48
	Pt	RS	**-0.11**	-0.10	-0.75	-0.71	0.22	0.23	0.22	**0.19**	0.49	0.51
		TS	**-0.07**	-0.11	-0.72	-0.71	0.25	0.24	0.24	**0.13**	0.50	0.51
	Cu	RS	**-0.11**	-0.10	-0.75	-0.79	0.21	0.21	0.21	**0.17**	0.47	0.48
		TS	**-0.21**	-0.12	-0.72	-0.77	0.21	0.21	0.21	**-0.32**	0.47	0.49
	Ag	RS	**-0.10**	-0.09	-0.75	-0.77	0.20	0.21	0.20	**0.18**	0.48	0.48
		TS	**-0.06**	-0.13	-0.68	-0.75	0.21	0.23	0.22	**-0.35**	0.50	0.48
	Au	RS	**-0.13**	-0.12	-0.71	-0.70	0.19	0.18	0.18	**0.17**	0.48	0.47
		TS	**0.03**	-0.18	-0.64	-0.68	0.21	0.20	0.20	**-0.19**	0.50	0.48

Supplementary Table 6. Pearson correlation coefficient (PCC) between descriptors used in this study. It has a value between +1 and −1, where +1 is total positive linear correlation, 0 is no linear correlation, and −1 is a total negative linear correlation. $\Delta G^{act,gas}$ shows the free energy of activation in vapor phase, H-bond denotes the change in mean of total hydrogen bonding (acceptor + donor) going from RS to TS, MC (molecular charge-transfer) represents the change in the absolute sum of partial charges on the reacting moiety going from RS to TS, and finally BC (cleaving-bond charge-transfer) expresses the change in sum of partial charges on the cleaving bond going from RS to TS (see Table 2 in the main text for the value of descriptors).

**Cleavage**	**MC, BC**	**H-bond, MC**	**H-bond, BC**	**H-bond, $\Delta G^{act,gas}$**	**BC, $\Delta G^{act,gas}$**	**MC, $\Delta G^{act,gas}$**
**O-H**	-0.99	0.83	-0.78	0.56	-0.67	0.65
**C-H**	-0.96	0.66	-0.43	-0.54	-0.45	0.22

Supplementary Table 7. Data of a linear model, $(y - \bar{y}) = \alpha_1(f_1 - \bar{f_1}) + \alpha_2(f_2 - \bar{f_2})$, for estimating the solvent effect ($\Delta\Delta G^{act} = \Delta G^{act,liq} - \Delta G^{act,gas}$), computed by *eSMS*, on O-H and C-H bond cleavages of ethylene glycol at 423 K. We have not considered the combination of MC and BC since they are totally correlated (see Table S7). The fits are based on 6 data points for each cleavage which correspond to six transition metals. Also, MAE represents the mean of absolute errors associated with the fit, in eV, defined as the difference between estimated and *eSMS* values.

	**O-H cleavage**			**C-H cleavage**		
$\boldsymbol{f_1, f_2}$	$\boldsymbol{\alpha_1}$	$\boldsymbol{\alpha_2}$	MAE	$\boldsymbol{\alpha_1}$	$\boldsymbol{\alpha_2}$	MAE
$\Delta G^{act,gas}$, BC	0.13	0.27	0.14	-0.04	0.54	0.03
$\Delta G^{act,gas}$, MC	0.06	-0.03	0.14	-0.10	-0.48	0.04
$\Delta G^{act,gas}$, H-bond	0.02	0.10	0.14	-0.27	-0.44	0.04
H-bond, BC	0.43	0.48	0.12	0.03	0.61	0.04
H-bond, MC	0.30	-0.15	0.14	0.19	-0.69	0.04

Supplementary Table 8. Data of a quadratic model, $(y - \bar{y}) = \alpha_1(f_1 - \bar{f_1}) + \alpha_2(f_2 - \bar{f_2}) + \alpha_3(f_1 - \bar{f_1})^2 + \alpha_4(f_2 - \bar{f_2})^2$, for estimating the solvent effect ($\Delta\Delta G^{act} = \Delta G^{act,liq} - \Delta G^{act,gas}$), computed by *eSMS*, on O-H bond cleavage of ethylene glycol at 423 K.

$\boldsymbol{f_1, f_2}$	$\boldsymbol{\alpha_1}$	$\boldsymbol{\alpha_2}$	$\boldsymbol{\alpha_3}$	$\boldsymbol{\alpha_4}$	MAE
$\Delta G^{act,gas}$, BC	-0.08	0.49	0.92	-4.38	0.02
$\Delta G^{act,gas}$, MC	-0.18	-0.44	1.29	-2.11	0.01
$\Delta G^{act,gas}$, H-bond	0.11	0.44	-0.44	2.12	0.08
H-bond, BC	0.56	0.59	1.82	-2.68	0.04
H-bond, MC	0.90	-0.62	2.26	-1.19	0.04

Supplementary Table 9. Solvent effects on the free energy of activation ($\Delta\Delta G^{act} = \Delta G^{act,liq} - \Delta G^{act,gas}$) of O-H and C-H bond cleavage of ethylene glycol on the (111) facet of six transition metal surfaces using the *iSMS* solvation scheme. Note that calculations were performed with the help of the COSMO-RS package with three different metal cavity radii: with default value (2.2230 Å for all transition metal elements), with a 10% increased value (2.4453 Å) and a 10% decreased (2.0007 Å) value relative to the default.

		**default**	**+10%**	**-10%**
**O-H cleavage**	**Ni**	-0.08	-0.06	-0.15
	**Cu**	-0.09	-0.08	-0.09
	**Ag**	-0.06	-0.06	-0.08
	**Pt**	-0.06	-0.05	-0.12
	**Pd**	0.03	-0.01	0.02
	**Au**	-0.03	0.00	-0.05
**C-H cleavage**	**Ni**	-0.04	-0.04	-0.04
	**Cu**	-0.12	-0.09	-0.15
	**Ag**	-0.03	-0.03	-0.05
	**Pt**	-0.09	-0.12	-0.15
	**Pd**	-0.01	-0.06	0.00
	**Au**	-0.17	-0.19	-0.08

Supplementary Table 10. Average fraction of TIP3P water molecules with different orientations (see Supplementary Figure 3 for explanation of the different orientations) within the first water layer of the surface (see first peak in height distribution function of the water O atom in Supplementary Figure 4). The data indicate that water orients itself similar across the metal surfaces and ~68% of them are in "parallel" orientation, ~23% in H-up orientation, and only 9% in H-down orientation (which lets the water hardly form hydrogen bonds with the condensed phase).

**Water Orientation**	**Ni**	**Cu**	**Ag**	**Pt**	**Au**	**Pd**
**Parallel**	68.17±0.07	68.10±0.40	67.85±0.11	68.08±0.29	68.25±0.12	67.93±0.25
**H-up**	22.93±0.10	23.15±0.25	22.78±0.07	23.05±0.15	22.63±0.07	22.81±0.26
**H-down**	8.90±0.16	8.75±0.17	9.38±0.07	8.87±0.17	9.12±0.12	9.26±0.15

Figures

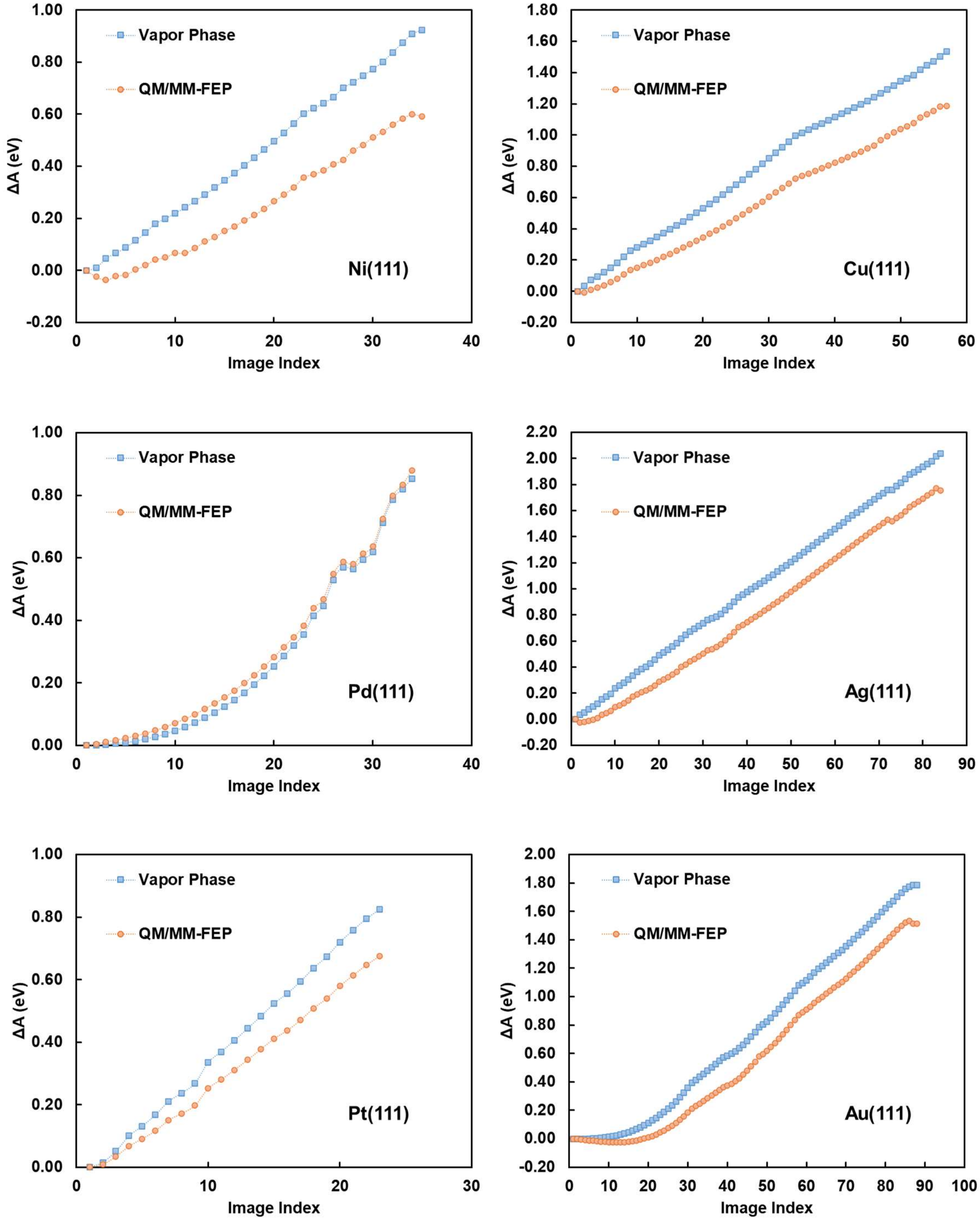

Supplementary Figure 1. Free-energy profiles for C-H bond cleavage of ethylene glycol in vapor and aqueous phases over the (111) facet of six transition metal surfaces at 423 K without considering vibrational contributions to the partition function. See Table 1 for corresponding data that include vibrational contributions. The aqueous phase profile portrays the average of three or more independent *eSMS*

calculations possessing 95% confidence intervals smaller than ±0.05 eV. The analogous plot for O-H bond cleavage is provided in the main text.

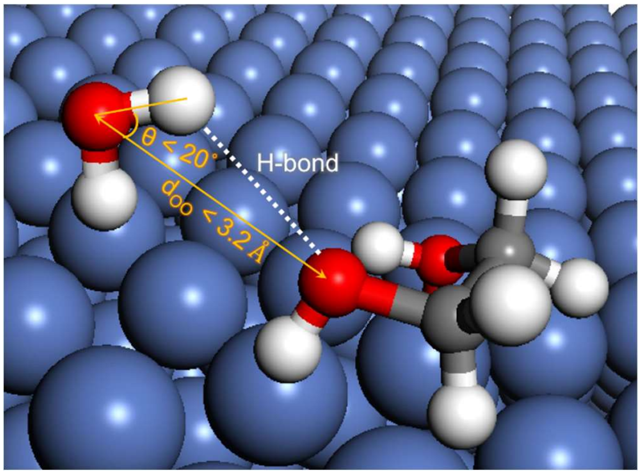

Supplementary Figure 2. Graphical representation of a geometric hydrogen bond definition used in this study. In the picture shown, water is the donor of hydrogen bonding, and ethylene glycol is the acceptor of hydrogen bonding.

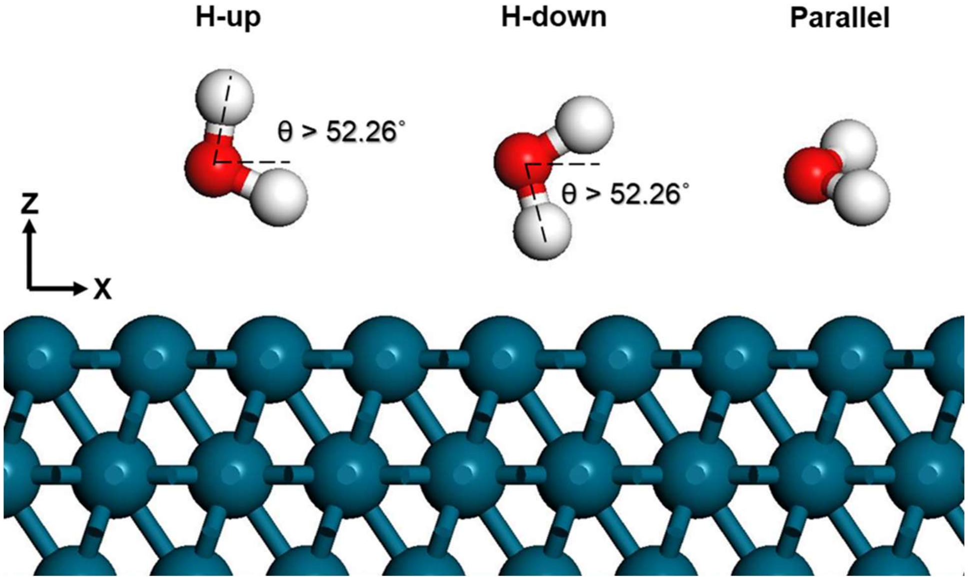

Supplementary Figure 3. Graphical representation of criterion used to distinguish different water orientations at the surface. A water molecule in the first layer next to the surface is considered in H-up conformation if the difference in z-coordinate of the H and O atom is larger than half the H-H distance of the water molecule; this corresponds for TIP3P water to an angle θ show above larger than 52.26º. Water molecules that are not considered H-up or H-down are labeled "parallel".

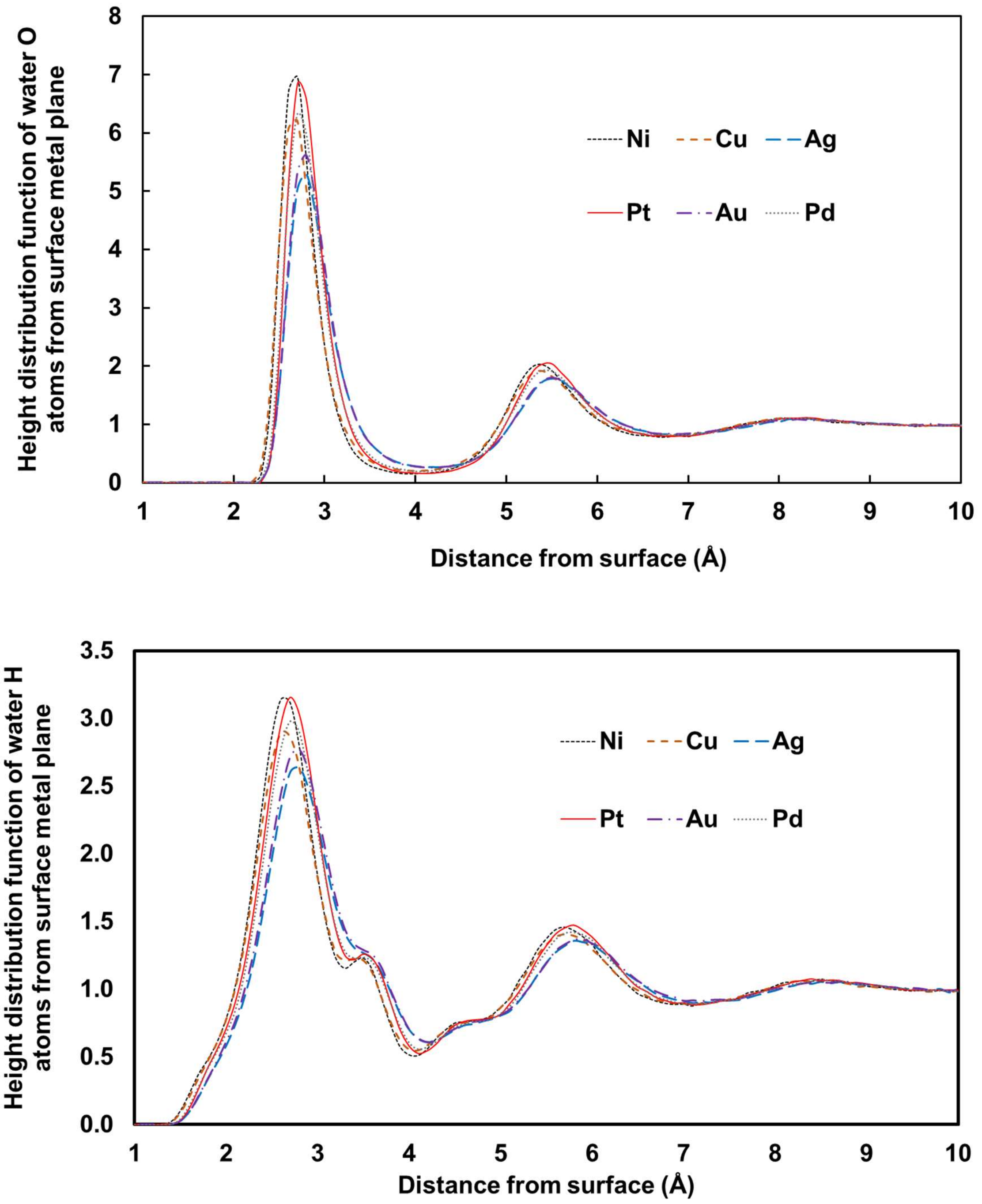

Supplementary Figure 4. Height distribution function of water O and H over (111) facet of six transition metal surfaces.

References:

1. Reed AE, Weinstock RB, Weinhold F. Natural-Population Analysis. *J Chem Phys* **83**, 735-746 (1985).
