# Supplementary material for: Dependency of solvation effects on metal identity in surface reactions
Source: Commun Chem. 2020 Dec 10;3:187. doi: 10.1038/s42004-020-00428-4 (PMC9814277; doi:10.1038/s42004-020-00428-4)
Supplement: Supplementary file 2 — Description of Additional Supplementary Files [file 42004_2020_428_MOESM2_ESM.pdf]

Description of Additional Supplementary Files

File Name: Supplementary Data 1
Description: Atomic coordinates of optimized clusters of reactant and transition states of O-H and C-H bond cleavages of ethylene glycol over (111) facet of six transition metals (Ni, Cu, Ag, Pd, Pt, Au) in gas and liquid phases.
